# Divergent Roles of the Auxin Response Factors in Lemongrass (*Cymbopogon flexuosus* (Nees ex Steud.) W. Watson) during Plant Growth

**DOI:** 10.3390/ijms25158154

**Published:** 2024-07-26

**Authors:** Guoli Wang, Jian Zeng, Canghao Du, Qi Tang, Yuqing Hua, Mingjie Chen, Guangxiao Yang, Min Tu, Guangyuan He, Yin Li, Jinming He, Junli Chang

**Affiliations:** 1 Guangdong Provincial Key Laboratory of Utilization and Conservation of Food and Medicinal Resources in Northern Region, School of Biology and Agriculture, Shaoguan University, Shaoguan 512005, China; wgl_d2022@hust.edu.cn (G.W.); zengjian@sgu.edu.cn (J.Z.); 2 The Genetic Engineering International Cooperation Base of Chinese Ministry of Science and Technology, Key Laboratory of Molecular Biophysics of Chinese Ministry of Education, College of Life Science and Technology, Huazhong University of Science and Technology, Wuhan 430074, China; dch2021@hust.edu.cn (C.D.); d202380961@hust.edu.cn (Q.T.); cmj@hust.edu.cn (M.C.); ygx@hust.edu.cn (G.Y.); hegy@hust.edu.cn (G.H.); yinli2021@hust.edu.cn (Y.L.); 3 Hubei Technical Engineering Research Center for Chemical Utilization and Engineering Development of Agricultural and Byproduct Resources, School of Chemical and Environmental Engineering, Wuhan Polytechnic University, Wuhan 430023, China; cyly122815@163.com (Y.H.); 12739@whpu.edu.cn (M.T.)

**Keywords:** ARF transcription factors, Cymbopogon, expression analysis, gene duplication, RNA-seq

## Abstract

Auxin Response Factors (ARFs) make up a plant-specific transcription factor family that mainly couples perception of the phytohormone, auxin, and gene expression programs and plays an important and multi-faceted role during plant growth and development. Lemongrass (*Cymbopogon flexuosus*) is a representative Cymbopogon species widely used in gardening, beverages, fragrances, traditional medicine, and heavy metal phytoremediation. Biomass yield is an important trait for several agro-economic purposes of lemongrass, such as landscaping, essential oil production, and phytoremediation. Therefore, we performed gene mining of *CfARFs* and identified 26 and 27 CfARF-encoding genes in each of the haplotype genomes of lemongrass, respectively. Phylogenetic and domain architecture analyses showed that CfARFs can be divided into four groups, among which groups 1, 2, and 3 correspond to activator, repressor, and ETTN-like ARFs, respectively. To identify the CfARFs that may play major roles during the growth of lemongrass plants, RNA-seq was performed on three tissues (leaf, stem, and root) and four developmental stages (3-leaf, 4-leaf, 5-leaf. and mature stages). The expression profiling of *CfARFs* identified several highly expressed activator and repressor *CfARFs* and three *CfARFs* (*CfARF3*, *18*, and *35*) with gradually increased levels during leaf growth. Haplotype-resolved transcriptome analysis revealed that biallelic expression dominance is frequent among *CfARFs* and contributes to their gene expression patterns. In addition, co-expression network analysis identified the modules enriched with *CfARFs*. By establishing orthologous relationships among *CfARFs*, sorghum *ARFs*, and maize *ARFs*, we showed that *CfARFs* were mainly expanded by whole-genome duplications, and that the duplicated *CfARFs* might have been divergent due to differential expression and variations in domains and motifs. Our work provides a detailed catalog of *CfARFs* in lemongrass, representing a first step toward characterizing *CfARF* functions, and may be useful in molecular breeding to enhance lemongrass plant growth.

## 1. Introduction

Phytohormones play central roles in the coordination of plant growth, development, and environmental responses. Auxin is a classical phytohormone that is required for plant growth, organ formation, tissue patterning, and cell elongation and proliferation [1,2]. Importantly, the typical nuclear auxin perception and signaling pathway is relatively conserved in higher plant species, and its evolution and conservation have been recently reviewed [3,4,5].

The classical auxin signaling pathway is composed of three components: the auxin nuclear receptor family, namely Transport Inhibitor 1/Auxin Signaling F-box (TIR1/AFB); the co-receptors encoded by the *Auxin*/*Indole-3-acetic acid* (*Aux*/*IAA*) gene family; and the Auxin Response Factor (ARF) family of transcription factors [2]. Auxin is perceived by the TIR1/AFB receptor and acts as a signaling molecule to promote the interactions between TIR1/AFB and Aux/IAA proteins, leading to the ubiquitination and degradation of Aux/IAA. Aux/IAA is a family of transcriptional co-repressors that directly interacts with and represses the transactivation activities of ARF transcription factors. With increased levels of auxin and degradation of Aux/IAA proteins, ARFs are released to induce the auxin response and regulate downstream gene expression [6].

With the expansion and complexation of the auxin signaling pathway during the evolution of higher plant species, distinct auxin-responsive transcriptional regulations specific to different tissue and/or cell types are thought to have contributed to the expansion of the *ARF* family and interactions between ARFs and other proteins [7,8,9]. Because of their functional importance in regulating auxin transcriptional responses, the structures, domain architectures, and transcriptional targets of ARF proteins have been extensively studied [10,11,12,13]. Phylogenetic studies from multiple species have revealed three evolutionarily conserved clades (namely clades A, B, and C), which is largely in agreement with the results of reporter gene assays [3]. Clade A ARFs are activators, while clade B ARFs are repressors, and clade C ARFs show no reporter gene expression. A typical ARF protein is generally separated into the N-terminal DNA-binding domain (DBD), C-terminal Phox and Bem1p (PB1) domain, and a middle region that is important for activator ARFs. The PB1 domain is highly homologous to motifs III and IV of Aux/IAA proteins. Both PB1 and the N-terminal dimerization domains (designated as DD1 and DD2) flanking the B3 DNA-binding domain are required for the homo- and/or hetero-oligomerization of ARFs and interactions with Aux/IAA proteins [6,11,13,14].

Our current knowledge of auxin signaling and the biological functions of auxin signaling components comes from extensive genetic and biochemical studies of model plant species, such as *Arabidopsis thaliana* and *Physcomitrium patens* [15,16,17,18,19]. By contrast, genetic and functional genomic research on ARFs from other agriculturally important crops are relatively limited, partly due to the functional redundancy between ARFs. ARF research in maize is a promising area with many remaining knowledge gaps [20,21,22,23,24,25], while sorghum (*Sorghum bicolor*) ARFs have recently undergone genome-wide characterization [26,27].

Cymbopogon is a genus of the family Poaceae, which contains ~180 species and is evolutionarily important as it represents one of the three related Panicoideae subtribes (the Anthistiriinae subtribe, with the others being the Sorghinae and Saccharinae subtribes, with sorghum and sugarcane as the representative species, respectively) [28,29,30,31]. The Cymbopogon genus includes many fragrant species that produce large amounts of essential oils with known chemo-diversity among species. Additionally, Cymbopogon species have wide applications in garden landscapes, as food ingredients, in beverages, fragrances, and cosmetics, as traditional medicines, and in phytoremediation for heavy metal pollution [28,29,30]. Biomass yield is an important trait for several agro-economic uses of Cymbopogon plants, such as gardening, essential oil production, and phytoremediation. However, molecular insights into biomass production are scarce for Cymbopogon species due to the lack of high-quality reference genomes for any Cymbopogon species. Recently, a set of genome sequences for *Cymbopogon citratus* based on Illumina short-reads was reported, containing ~20,000 unassembled contigs [32]. To address which gene members of the auxin signaling components may contribute to plant growth and biomass accumulation in Cymbopogon plants, we chose lemongrass (*Cymbopogon flexuosus*, diploid, 2n = 20) and the *ARF* gene family as our research focus for the following reasons [28,29]. First, lemongrass has a small genome size (approximately 700 Mb) and heterozygosity rate (1.44%), allowing us to generate the first high-quality, chromosome-level reference genome in the Cymbopogon genus (https://doi.org/10.6084/m9.figshare.25283638.v1 (accessed on 20 January 2024)) [33]. Second, lemongrass usually reproduces asexually and has a growth period in which it produces multiple tillers, with leaves sprouting and elongating in each tiller. Auxin signaling and transcriptional responses likely play a role during this growth period. Third, lemongrass is evolutionarily related to sorghum (*Sorghum bicolor*), maize (*Zea mays*), and rice (*Oryza sativa*). On the one hand, these monocot grass species have all experienced ancient whole-genome duplication events [34,35], with the sorghum–maize gene orthologous relationships well established [36]. On the other hand, functional knowledge of ZmARFs and OsARFs serves as a valuable resource for the comparative functional genomics of CfARFs [2].

With the above-mentioned considerations and our haplotype-resolved lemongrass genome, the present study aims to characterize *CfARF* genes in a haplotype genome-wide manner to obtain insight into the expansion and divergence of *CfARFs* in order to identify *CfARF* candidates that likely play important roles during the growth of lemongrass plants. In the present study, we provide a comprehensive catalog of CfARFs, serving as the starting point for deepening the molecular understanding of auxin-regulated gene expression in lemongrass; demonstrate that whole-genome duplication and tandem duplication both contributed to the expansion of CfARFs; and suggest that, after the whole-genome duplication event of the Poaceae common ancestor, duplicated CfARF pairs may possibly have become divergent in ways that differed from OsARFs. Furthermore, the transcriptomic analysis highlights that biallele expression bias is frequently adopted by CfARFs as an approach to potentially exert divergent functions during the growth of lemongrass.

## 2. Results and Discussion

### 2.1. A Catalog of CfARFs in the Lemongrass Genome

In the lemongrass genome, 26 and 27 genes encoding CfARF proteins were identified in the haplotype 1 and 2 genomes (abbreviated as hap1 and hap2 hereafter), respectively (Table 1). These identified genes contained 25 *CfARFs* allelic pairs and one hap1-specific *CfARF* (*CfARF22*) and two hap2-specific *CfARFs* (*CfARF10b* and *CfARF31b*).

**Figure 1:**
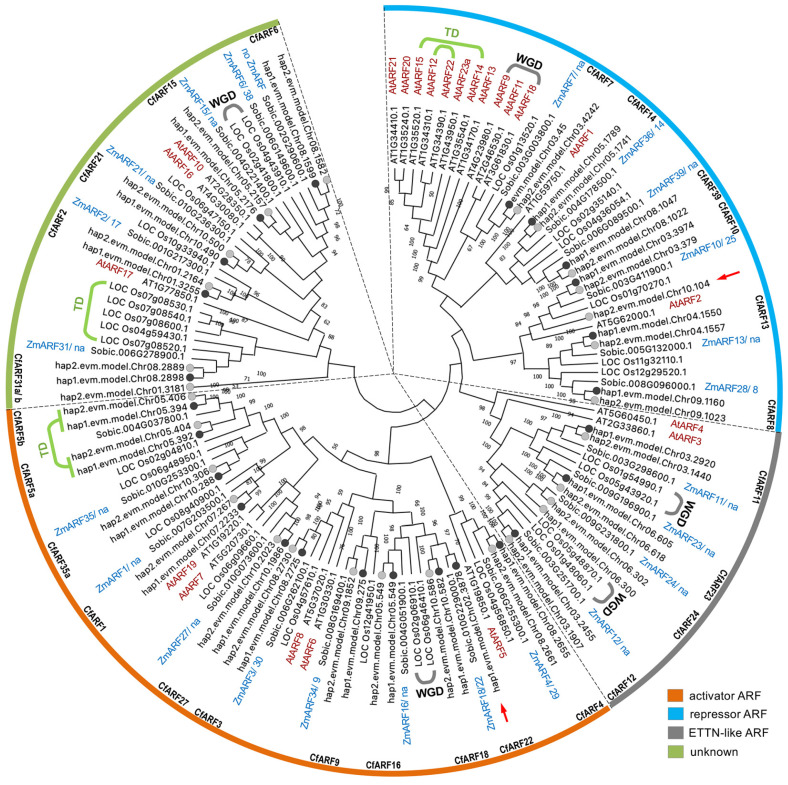
Phylogenetic analysis of the CfARF family. CfARFs, SbARFs, OsARFs, and AtARFs were used in the phylogenetic analysis, with *ZmARFs* orthologs annotated next to the corresponding *SbARF* geneIDs in the phylogenetic tree. *CfARFs* from the lemongrass hap1 and hap2 genomes are indicated with black and grey dots, respectively, on the tree branches. The four phylogenetic groups of CfARFs (i.e., groups 1, 2, 3, and 4) are indicated in the colored outermost circle in blue, orange, gray, and green, respectively. CfARF proteins specifically encoded by the lemongrass hap1 or hap2 genome are indicated with red arrowheads. *OsARFs* and *AtARFs* expanded due to ancient WGD events in monocots and dicots, respectively, are labeled with gray brackets. *ARF* genes expanded due to tandem duplication events (TD) are labeled with light green brackets.

ARF proteins represent one of the core components of the canonical nuclear auxin signaling pathway and are evolutionarily conserved in higher plant species. According to transactivation assay results and their domain architectures, ARFs are classified into clade A, clade B, and clade C [3]. Generally, clade A and B ARFs are activators and repressors, respectively, while clade C ARFs are much less well studied in terms of their functions in auxin signaling, transcriptional activity, and involved biological processes [3]. Here, CfARFs encoded by the genes from the two haplotype genomes fell into four phylogenetic groups (Figure 1), for which groups 1 and 2 corresponded to clade B and A ARFs, respectively. Group 3 CfARFs were the ETTIN-like group of ARF proteins, while group 4 CfARFs were homologous to the rest of the clade C ARFs; there is not much knowledge regarding their molecular functions. Thus, group 3 and 4 CfARFs corresponded to clade C ARFs [20,36]. The phylogenetic analysis was of high quality. Firstly, the best reciprocal blast hits of SbARFs were clustered together with the corresponding CfARFs. Secondly, orthologous OsARFs and SbARFs were within the same phylogenetic branches based on the established rice–sorghum gene orthologous relationships [34,36,37] (Figure 1). Thirdly, all of the CfARFs encoded by allelic paired genes were clustered within the same phylogenetic branches, and these CfARFs had high protein similarity (more than 98%), while the protein similarities between CfARFs encoded by non-haplotypic genes ranged from ~25% to 80% (Figure S1).

The expansion and contraction of gene families, particularly those exerting regulatory roles (e.g., transcription factors), are important for the divergence and redundancy of gene functions and have contributed to shaping the complex molecular networks and traits during plant evolution. Whole-genome duplication (WGD), tandem duplication (TD), and fragmental duplication (FD) are among the major approaches for gene expansion. In particular, the Arabidopsis lineage has experienced multiple ancient WGD events (namely α-, β-, and γ-WGDs), while a polyploidization event (ρ-WGD) occurred ~70 million years ago (MYA) to the monocot ancestor, which further evolved into numerous grass species, including rice, wheat, maize, sorghum, and Cymbopogon [34,38,39]. The *AtARFs* duplicated by WGD and TD events were identified using these pieces of evolutionary information, demonstrating that both WGD and TD contributed to the expansion of *AtARFs* (Figure 1). By contrast, WGD may have played a more important role than TD in the expansion of *OsARFs*. Four pairs of paleoduplicated *OsARFs* were detected and one locus of *OsARF* was expanded into four copies (LOC_Os07g08520, LOC_Os07g08530, LOC_Os07g08540, and LOC_Os07g08600) due to TD. All of the WGD-derived duplicates were conservatively maintained in sorghum and lemongrass, whereas the *ARF* genes orthologous to TD-derived *OsARFs* were not duplicated in sorghum and lemongrass. In addition, *CfARF5a* and *CfARF5b* were likely expanded by a TD specific to the lemongrass genome. Therefore, the WGD events experienced in the ancestry of Poaceae could be the major driver of *ARF* expansion in these grass species.

**Figure 2:**
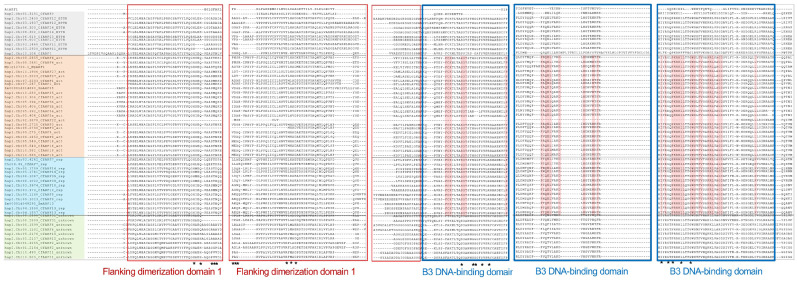
Conservation of the CfARF DNA-binding domain. Amino acid alignment of the DNA-binding domain of CfARFs was performed with representative activator and repressor ARFs (MpARF1 and ZmARF35 as activators, AtARF1 and ZmARF13 as repressors). The phylogenetic clades for CfARFs are indicated in background colors (orange, light blue, gray, and green meaning groups 1, 2, 3, and 4, respectively). B3 DNA-binding domain (DBD) is indicated by the blue box, while flanking dimerization domains 1 and 2 are indicated by the red and gray boxes, respectively. The sequences of flanking dimerization domains 1 and 2 were adopted from those previously described [9,12,40]. Asterisks in the B3 DBD region indicate DNA-contacting residues described for AtARF1 [12]. Asterisks in the flanking dimerization domains indicate residues at the ARF dimer interface [14]. Amino acid residues conserved in the aligned ARFs for either group 1 or group 2 ARFs are highlighted with red background color.

**Figure 3:**
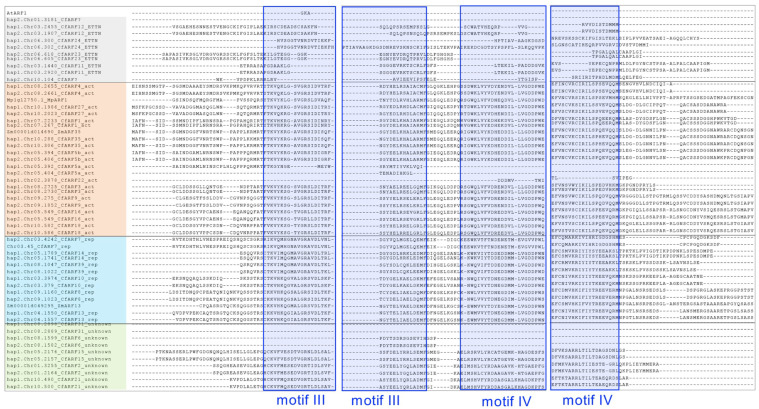
Conservation of the CfARF C-terminal region. Amino acid alignment of the DNA-binding domain of CfARFs was performed with representative activator and repressor ARFs (MpARF1 and ZmARF35 as activators, AtARF1 and ZmARF13 as repressors). The phylogenetic clades for CfARFs are indicated in background colors, as described in Figure 2. The amino acid sequences similar to Aux/IAA motif III or IV are indicated in blue boxes.

Owing to the importance of ARF proteins in the auxin signaling pathway, extensive knowledge has been developed on their structure–function relationships. Through alignment with representative activator and repressor ARFs (MpARF1 and ZmARF35 as activators, ZmARF13 as a repressor), we identified the domains and motifs in CfARFs that are important for exerting ARF functions (Figure 2, Figure 3 and Figures S2 and S3). Almost all of the activator and repressor CfARFs contained the conserved B3 DNA-binding domain in their N-terminal regions, except for CfARF22, suggesting the potential loss of the DNA-binding ability of CfARF22 (Figure 2 and Figure S2). In the N-terminal region, two dimerization domains flanking the B3 DNA-binding domain are important for ARF homodimerization and heterodimerization [9,12,40], of which the key amino acid residues likely located at the protein dimer interface are labeled in Figure 2 and Figure S2. These flanking dimerization domains provide an additional layer of DNA-binding regulation toward the tandem repeat Auxin Response Elements (AuxREs) [12,41,42]. CfARF22 lacked the conserved sequence of flanking dimerization domain 1 and had only a partial sequence of flanking dimerization domain 2. The middle region of ARF proteins is critical for their transcriptional activation or repression ability, with the Q-rich stretches in activator ARFs conferring transactivation activity [11,43]. Indeed, Q-rich stretches that varied in length were only identified in activator CfARFs, while CfARF4 (hap1.chr8.2655 and hap2.chr8.2661), CfARF5a (hap2.chr5.404), and CfARF22 (hap1.chr2.3878) lacked the typical Q-rich stretches (Figure S3). In the C-terminal region, the PB1 domain showed high sequence similarity with motifs III and IV of Aux/IAA, which allow ARF homo- and hetero-oligomerization and/or direct interactions with Aux/IAA proteins [11]. Conserved sequences of motifs III and IV were detected in most activator and repressor CfARFs, as well as in group 4 CfARFs, with a few activator and group 4 CfARFs lacking motifs III and IV. Taken together, the detailed analysis of domain architectures of CfARFs revealed somewhat divergent sequences in the domains and motifs that are important for ARF molecular functions.

### 2.2. Expression Patterns of CfARFs during the Plant Growth of Lemongrass

**Figure 4:**
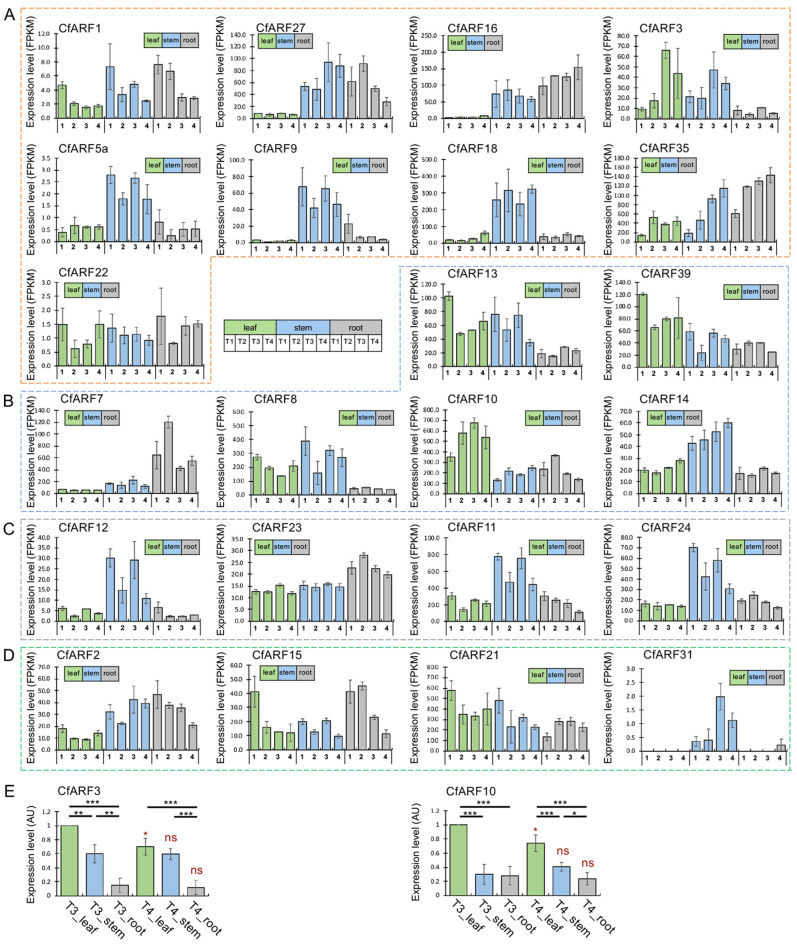
Expression patterns of *CfARFs* in the leaf, stem, and root tissues during the four lemongrass growth stages. The expression patterns of *CfARFs* belonging to phylogenetic groups 1, 2, 3, and 4 are given for the four stages and three tissues (**A**, **B**, **C**, and **D**, respectively). To distinguish the CfARFs from groups 1, 2, 3, and 4, there figures are surrounded by orange, blue, grey and green broken lines, respectively. The leaf, stem, and root tissues are indicated in green, blue, and gray, respectively. The four stages are labeled as 1, 2, 3, and 4, and described in the Materials and Methods Section 3.5. “Transcriptomic of the lemongrass and coexpression network analysis”. (**E**) Quantitative RT-PCR validation of the expression of *CfARF3* and *CfARF10* at the T3 and T4 stages. qPCR was performed with three biological replicates, with the statistical difference determined by Student’s *t*-test (*, *p* < 0.05; **, *p* < 0.01; ***, *p* < 0.005). Within each gene and stage, statistical differences are indicated with black asterisks, while statistical differences in the expression levels from the same tissues between the T3 and T4 stages are shown with red asterisks, with “ns” indicating “not significant”. *CfActin* (geneID: hap1.evm.model.Chr01.4294) was used as the internal reference gene for qPCR with all of the qPCR primers provided in Table S2.

To explore the potential functions of CfARFs in plant growth, we profiled the expression patterns of *CfARF*s in four developmental stages and three tissues (i.e., leaf, stem, and root) by performing RNA-seq analysis (Figure S4; Table S1). Among the 26 haplotypic pairs of *CfARF*s, 23 were expressed in the sampled tissues, with most *CfARF*s expressed ubiquitously (Figure 4). Notably, the expression levels varied dramatically between *CfARF*s. For example, *CfARF16*, *18*, and *35* represented highly expressed activator *ARF*s, with *CfARF3*, *9*, and *27* being intermediately expressed. By contrast, the expression levels did not vary as extensively among repressor *CfARF*s as was observed among activator *CfARF*s, with *CfARF10* being the most highly expressed in all three tissues. For ETTN-like and group 4 *CfARFs*, most *CfARF* genes were widely expressed in the leaf, stem, and root, except *CfARF31*, which was uniquely expressed in the stem. The expression preference among tissues was detected for some *CfARFs*. For instance, *CfARF3* was gradually upregulated and had apparently higher expression levels in the leaf and stem than in the root tissue. *CfARF9* exhibited higher expression levels in the stem than in the leaf and root. *CfARF16* and *CfARF27* showed high expression levels in the root and stem, with low expression levels in the leaf. In addition, *CfARF18* was particularly highly expressed in the stem. The significantly increased expression levels for some activator *CfARFs* (e.g., *CfARF3*, *18*, and *35*) were not obviously seen for other groups of *CfARFs*. The ubiquitous and relatively stable expression patterns of many *CfARF*-encoding genes suggest that CfARFs within a phylogenetic group could be functionally redundant, and some CfARFs might play multi-faceted roles during plant growth. To further validate our RNA-seq expression analysis results, quantitative reverse-transcribed PCR (qRT-PCR) was performed for *CfARF3* and *CfARF10*, as these two *CfARF* genes had gradually increased expression levels during leaf maturation and had particularly higher expression in the leaf than in the root. Indeed, the qRT-PCR expression results showed that *CfARF3* and *10* had the highest expression levels in the leaf, followed by the stem and root (Figure 4E; Table S2), suggesting that they might have functions involved in leaf growth and stem expansion or elongation.

**Figure 5:**
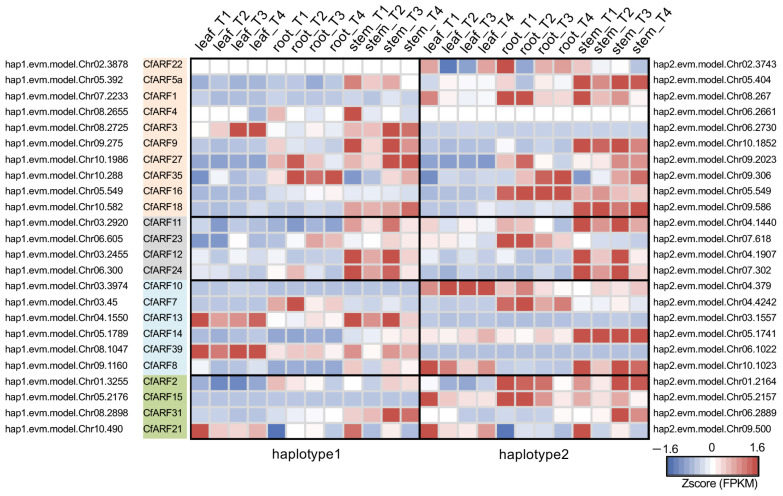
Allelic expression patterns of *CfARFs*. The allelic expression profiles of each pair of *CfARF* haplotypes are shown in the heat map, with the colors indicating the Z score of the expression level (in FPKM) and the number on each grid indicating the FPKM expression level. *CfARF* genes are sorted into phylogenetic groups.

We took advantages of our haplotype-resolved lemongrass genomes to investigate whether expression bias could exist between *CfARF* alleles. Within the 23 expressed bialleles of *CfARFs*, 14 allelic pairs had biallelic expression dominance (BED) (Figure 5). BED appeared to be a common expression phenomenon for *CfARFs*, as it was seen in *CfARFs* from all four phylogenetic groups. In particular, BED expression patterns were detected only in certain stages and tissues for five *CfARFs* (i.e., *CfARF9*, *11*, *22*, *23*, and *24*). By contrast, the allelic expression analysis of ubiquitously expressed *CfARFs* (such as *CfARF1*, *CfARF3*, and *CfARF16*) identified the dominant alleles within a given *CfARF* allelic pair that contributed to a large portion or all of the expression level.

### 2.3. Co-Expression Analysis of CfARF Genes

**Figure 6:**
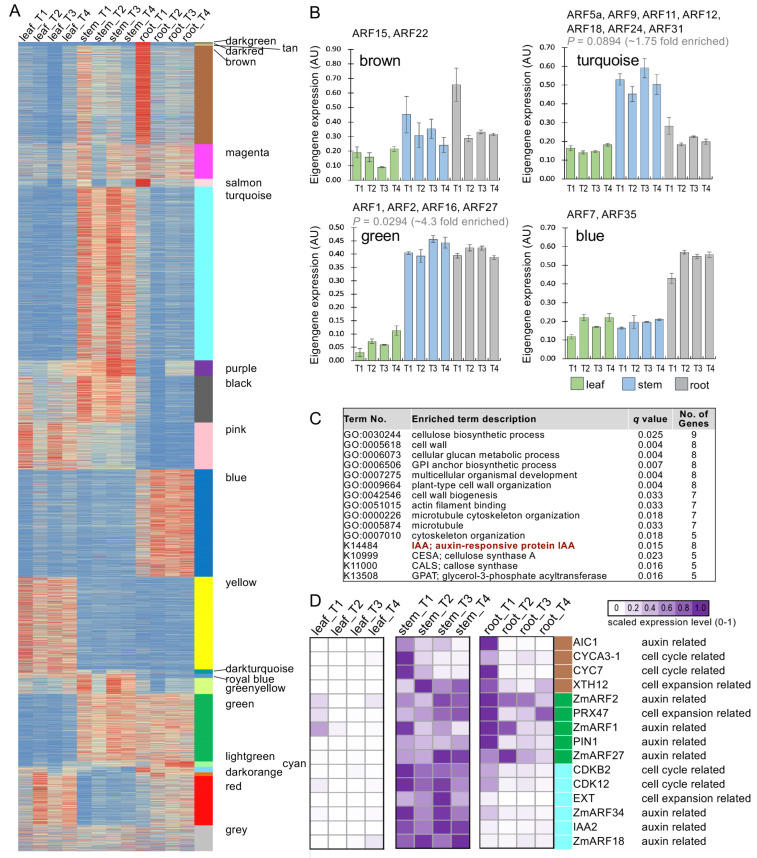
Co-expression network analysis-identified modules with *CfARFs* and other genes related to auxin signaling and cell growth. (**A**) The heat map showing the 21 co-expression modules identified with WGCNA. The module names are labeled and indicated with corresponding colors. (**B**) Representative expression patterns (eigengenes) of the four typical *CfARF*-containing modules, with the *CfARFs* labeled for each module. In particular, the green and turquoise modules appear to be enriched with *CfARFs*, as determined by the hypergeometric test (*p* < 0.05), while *P_hypergeometric_* for the turquoise module was close to the significant level (0.0894). (**C**) The enriched functional terms (including GO and KEGG) in the turquoise module, with the term no. and *q* values given. (**D**) In the brown, green, and turquoise modules, several genes homologous to the maize genes known to function in auxin biosynthesis, signaling, the cell cycle, and cell expansion were identified [44]. The heat map shows the relative expression of these genes in the leaf, stem, and root tissues of lemongrass, with their modules indicated with corresponding colors.

We performed weighted gene co-expression network analysis (WGCNA) for a total of 18,352 differentially expressed genes and constructed a gene network comprising 21 co-expression modules (Figure 6A and Figure S5). Since our aim was to obtain insight into *CfARFs* and their potential functions using RNA-seq-based network analysis, we focused on the *CfARF*-containing co-expression modules. *CfARFs* were found in the black, blue, brown, dark turquoise, green, pink, royal blue, turquoise, and yellow modules, whereas only a few modules hosted more than one *CfARF* gene, suggesting the non-random distribution of *CfARFs* in the co-expression modules (Figure 6). The representative expression patterns of CfARF-containing modules (i.e., the blue, brown, green, and turquoise modules) are visualized, showing higher expression levels in the stem and/or root than in the leaf tissue (Figure 6B). This expression feature allowed us to speculate that the *CfARFs* in these modules might be involved in the elongation and/or expansion of stem and root tissues. In addition, the green and turquoise modules appear to be enriched in ARF genes (Figure 6B). Since seven *CfARFs* were detected in the turquoise module, we performed functional enrichment analysis with the ClusterProfiler package. Indeed, the “auxin responsive IAA” term was enriched in the turquoise module, together with several functional terms related to the growth of stem and biomass accumulation (e.g., cell wall biogenesis, microtubule cytoskeleton organization, and cellulose biosynthetic process) (Figure 6C). All of the functional enrichment results of the 21 co-expression modules are provided, providing a starting point for investigating the molecular networks related to *CfARFs* (Table S3). Furthermore, we utilized previously known maize genes related to cell growth and auxin and discovered that these cell growth- and auxin-related genes (e.g., IAA2, CDKB2, CDK12, and PIN1) were co-expressed with several *CfARFs* in the brown, green, and turquoise modules (Figure 6D) [44]. Overall, these analyses indicated that several *CfARFs* might work with other genes related to auxin biosynthesis, signaling, the cell cycle, and cell expansion within the molecular networks of the green and turquoise modules.

### 2.4. Divergence of CfARFs

In the present study, four pairs of *CfARFs* (i.e., *CfARF11*-*CfARF23*, *CfARF12*-*CfARF24*, *CfARF16*-*CfARF18*, and *CfARF6*-*CfARF15*) were expanded due to ancient WGD events [33], while *CfARF5a* and *5b* were duplicated probably owing to lemongrass-specific TD events. These genes provide examples for us to examine whether *CfARFs* could become divergent, and, if so, what the mechanisms are that drive the divergence of *CfARFs*. CfARF11, CfARF23, CfARF12, and CfARF24 have conserved domain architectures without apparent variations in the protein sequence (Figure 2 and Figure 3). However, the duplicated CfARFs from distinct phylogenetic groups exhibited different similarities in their protein sequences (Figure S1). For example, WGD-derived activator ARFs CfARF16 and CfARF18 shared ~75% protein sequence identity. Similarly, the TD-derived activator ARF pair, CfARF5a and 5b, shared more than 78% sequence identity. By contrast, CfARF6 and 15 from phylogenetic group 4 shared 63% sequence identity. CfARF11 and 23 from phylogenetic group 3 (ETTN-like ARFs) shared only 48% of sequence identity, while the protein sequences of CfARF12 and 24 were ~56% identical with each other. These results indicated that the activator ARFs encoded by the duplicated gene pairs in lemongrass tended to have higher sequence similarity than those from other phylogenetic groups, suggesting the importance of maintaining conserved architectures of domains and motifs for these CfARFs.

In terms of expression, the ETTN group WGD-derived pairs, *CfARF11*-*CfARF23* and *CfARF12*-*CfARF24*, exhibited similar patterns, with high expression levels detected in the stem tissue (Figure 4), suggesting that *CfARF11-CfARF23* and *CfARF12-CfARF24* pairs may be functionally redundant. In group D CfARFs, CfARF6 and CfARF15 differed in conserved motifs: CfARF6 lacked motifs III and IV for interactions with Aux/IAA proteins (Figure 3). Moreover, *CfARF15* was ubiquitously expressed in the leaf, stem, and root, whereas *CfARF6* was not expressed. *CfARF6* might be expressed in certain organs or tissues (floral organs, for example) or be specifically responsive to some biotic and abiotic stresses. These results indicated the divergent roles of *CfARF6* and *CfARF15* during lemongrass growth and development. In addition, the *CfARF16-CfARF18* WGD pair exhibited distinct expression patterns, with *CfARF18* preferentially expressed in the stem and *CfARF16* expressed in both the stem and root (Figure 4). This suggests that *CfARF16* and *CfARF18* may become functionally divergent in the root. Similar expression divergence was also observed in the TD-duplicated *CfARFs*. *CfARF5a* was expressed in all three tissues with higher expression in the stem, whereas *CfARF5b* was not expressed.

Additionally, the ETTN-like ARFs appeared to be functionally important during plant growth and development. For instance, AtARF3/ETTIN is a master regulator of the morphogenesis of the female reproductive gynoecium [45,46]. AtARF3 is a conserved non-canonical ARF protein that senses and translates auxin levels into multiple transcriptome responses (including both the activation and repression of genes) [47]. While the functions of the ETTN-group ARF homologs remain to be investigated in major crops (e.g., rice and maize), our phylogenetic analysis demonstrated that the ETTN ARFs duplicated from the Poaceae ancestor were all kept in rice, sorghum, maize, and lemongrass (Figure 1). Moreover, taking advantage of previously reported information about the sorghum–maize orthologous relationships and maize paleo-tetraploidization, we found that maize-specific duplicate copies of ZmARF11, 12, 23, and 24, respectively, were probably deleted, implying the potential evolutionary importance of maintaining the four copies of ETTN-like ARFs in Poaceae species [36,48,49].

Rice is a model species of monocot crops for gene functional studies. Owing to the extensive available expression data of rice, we obtained some expression patterns of OsARFs, aiming to gain insight into several questions [50]: (1) to compare the divergence of gene expression within WGD-duplicated gene pairs; (2) to address whether the expression divergence of evolutionarily conserved WGD pairs evolved similarly or distinctly between the species. In rice, *OsETT1* (LOC_Os05g48870) and *OsETT2* (LOC_Os01g48060) were orthologous to *CfARF12* and *24*, respectively; *OsARF3* (LOC_Os01g54990) and *OsARF14* (LOC_Os05g43920) were orthologous to *CfARF11* and *23*, respectively. The rice genome includes a pair of WGD-derived activator *ARFs*, *OsARF6* (LOC_Os02g06910) and *OsARF18* (LOC_Os06g46410), and a pair of group 4 *ARF*s, *OsARF8* (LOC_Os02g41800) and *OsARF10* (LOC_Os04g43910) (Figure 1). The rice ARF nomenclature used herein is according to the IC4R and funRicegenes database [50,51]. While the sampling stages and tissues from the rice IC4R expression database were not directly comparable with those of the present lemongrass study, the rice expression database presents the tissue-preference patterns of gene expression by collecting and standardizing the expression data from numerous publications [50]. However, some of the tissues reported in the IC4R database (i.e., root, leaf, and shoot) could be comparable with the leaf, root, and stem tissues used in our lemongrass study. With this prerequisite, we observed that rice ETTN-like *ARF*s (*OsETT1*, *OsETT2*, *OsARF3*, and *OsARF14*) were highly expressed in the anther, leaf, panicle and seed tissues, with the four *OsARF*s exhibiting similar tissue preference of expression (Figure S6). *CfARF12* and *24* had the highest expression levels in the stem tissue, followed by the leaf and root, clearly showing that their expression patterns differed from the rice orthologs (*OsETT1* and *2*) (Figure 5). *CfARF11* and *23* were highly expressed in the stem tissue, followed the root and leaf tissues, with allelic expression bias detected for these two *CfARF*s. The tissue expression patterns of *CfARF11* and *23* were different from those of the *OsARF3* and *14*, which were highly expressed in the leaf (Figure 5 and Figure S6). Rice activators *OsARF6* and *18* were expressed mainly in the panicle and seed, followed by the leaf, root, and shoot. Th expression patterns of *OsARF6*/*18* were also distinct from those of lemongrass orthologs *CfARF16* and *18*. These examples well demonstrate that while the ARF gene family has been expanded in rice and lemongrass largely due to WGD, WGD-duplicated orthologs have evolved distinct expression patterns between rice and lemongrass, possibly due to different evolutionary constraints (e.g., environmental adaptation, human selection, and mode of propagation). In turn, our comparison between rice and lemongrass duplicated ARF orthologs well supports that gene functions cannot be simply inferred solely based on the evolutionary information of gene families and species and that comparisons of expression patterns and/or domain and motif architectures are indispensable in predicting the potential functions of gene members. To summarize, our analysis suggests that different expression patterns between each pair of WGD- or TD-derived *CfARF* genes could contribute to their functional divergence, while variations in the conserved domains and motifs may also be related to the divergence between *CfARF6* and *CfARF15*.

## 3. Materials and Methods

### 3.1. Plant Materials

Diploid lemongrass (*Cymbopogon flexuosus* (Nees ex Steud.) W. Watson) accession SG-01 was planted in the experimental fields of Shaoguan University (Shaoguan, China) and Huazhong University of Science and Technology (Wuhan, China), respectively.

### 3.2. Identification and Phylogenetic Analysis of CfARF Genes

To comprehensively identify the ARF proteins and their encoding genes, a combined approach was taken using both the BLAST-based method and the domain search method. Sorghum (*Sorghum bicolor*) ARF proteins in the BTx623 genome and rice (*Oryza sativa*) ARF proteins in the rice Nipponbare genome were retrieved based on previous reports and used for the BLASTp search to identify potential CfARF proteins (E-value < 1 × 10^−5^) [27,52]. In parallel, the hidden Markov model (HMM) profiles of the B3 domain (PF02362) and ARF domain (PF06507) were employed for the hmmsearch against all of the protein sequences of the lemongrass genome. The identified putative CfARF proteins from both search methods were combined and re-confirmed with domain analysis using the InterPro database (https://www.ebi.ac.uk/interpro/ (accessed on 30 March 2024)). This CfARF identification process was performed for both haplotype genomes of lemongrass.

The protein sequences of CfARFs were aligned using MUSCLE with default parameters. *Arabidopsis thaliana* ARFs (AtARFs), OsARFs, and SbARFs were used for protein sequence alignment and phylogenetic tree construction [27,42,52,53]. CfARFs were designated according to phylogenetic clades and neighboring SbARFs, for which the established orthologous relationships between sorghum and maize genes were used [36]. ZmARFs have been identified and designated, with some ZmARFs functionally investigated [20,22,25,44]. To identify those *ARF*-encoding genes that have evolved from whole-genome duplication (WGD) events, information regarding Arabidopsis ancient WGD events (i.e., α-, β-, γ-WGDs) and Poaceae WGD events (ρ-WGD) was adopted from previous studies [37,38].

A phylogenetic tree including AtARFs, OsARFs, SbARFs, and CfARFs was constructed using the maximum-likelihood (ML) method, with 1000 bootstrap replicates using MEGA-X software [54,55].

### 3.3. Analyses of the Molecular Properties of CfARF Proteins

The protein characteristics (i.e., protein length (aa), theoretical isoelectric point (pI), and molecular weight (Da)) were calculated using the Expasy ProtParam tool (www.expasy.org/resources/protparam (accessed on 30 March 2024)). The percentage of sequence identity between CfARF proteins was calculated using the Clustal-Omega tool on the EMDL-EBI database (https://www.ebi.ac.uk/Tools/msa/clustalo/ (accessed on 10 April 2024)), which generates the ‘Percent Identity Matrix’, showing the percentage of sequence identity between each pairwise comparison.

### 3.4. Sequence Analysis of CfARF Proteins

Detailed sequence analysis was performed to identify the protein domains, motifs, and key amino acid residues that are important for the functions of ARFs [9,12,13,40]. MpARF1 (Mp1g12750) and ZmARF35 (Zm00001d014690) were used as representative activator ARFs, while AtARF1 (AT1G59750) and ZmARF13 (Zm00001d049295) were used as representative repressor ARFs when performing the sequence analysis. The N-terminal DNA-binding domain (DBD) was further separated into the B3 DNA-binding domain and two flanking dimerization domains (DD1 and DD2), and key amino acid residues that were thought to be likely located on the dimer interface or those directly contacting with DNA were indicated in the alignment [12,39]. In the middle region (MR) of activator ARFs, Q-rich stretches were identified as the functionally important motif for trans-activation ability [11,42]. In the C-terminal region, the Phox and Bem 1 domain (PB1) acts as an oligomerization domain, allowing homo- and/or hetero-oligomerization with Aux/IAA proteins (cite). Motifs III and IV that show high homology to those of Aux/IAA proteins within the PB1 domain were also indicated in our sequence analysis.

### 3.5. Transcriptome of the Lemongrass and Coexpression Network Analysis

RNA-seq analysis was performed to characterize the expression of *CfARFs*, including three tissues (leaf, stem, and root) and four developmental stages (3-leaf stage, 4-leaf stage, 5-leaf stage, and mature stage, abbreviated as T1, T2, T3, and T4, respectively). Lemongrass plants of accession SG-01 were planted in the experimental field of Shaoguan University, using a randomized complete block design with three replicates to collect the tissue samples [56,57]. The four stages were selected as they represented the time course of leaf growth and biomass increase, which was suitable for identifying functional genes and regulators involved in this process. Each block was used to obtain samples of the four stages for biological replicates, and the sampling stage was randomly assigned to a plot within each block. Each plot consisted of ten rows, among which the peripheral two rows were not used for sampling to avoid the edge effect. Each row had eight lemongrass plants. The eight central rows were separated into four subplots (two rows for each subplot), corresponding to each sampling stage. When sampling, the central tillers of each plant with a similar size were tagged and used. All of the spikes were collected in the morning (between 9:00 A.M. and 11:00 A.M.) to avoid potential circadian influences on the transcriptome and metabolome. The central tillers from eight plants were collected from the field and were transferred to the lab and dissected immediately, followed by snap freezing in liquid nitrogen. Half of a leaf from the leaf top was collected as the leaf tissue from central tillers and the main stem (leaf sheath removed) was sampled, with root tissues sampled after cleaning with water. The sampled leaf, stem, and root tissues were subjected to transcriptomic analysis (RNA-seq).

Total RNA was extracted using TRIzol reagent (Invitrogen, Carlsbad, CA, USA). The quality of the extracted RNA samples was examined using agarose gel electrophoresis, the NanoDrop 2000 (Thermo Fisher Scientific, Inc., Carlsbad, CA, USA), and an Agilent 2100 Bio-analyzer (Agilent, Santa Clara, CA, USA). Standard protocols for the Illumina NovaSeq platform (Illumina, San Diego, CA, USA) were used for construction of the mRNA libraries. The RNA-seq libraries were sequenced to generate 150 bp paired-end reads. For sequence quality control, cutadapt (https://cutadapt.readthedocs.io/en/stable/ (accessed on 10 April 2024)) and FASTX-Toolkit (https://github.com/Debian/fastx-toolkit (accessed on 10 April 2024)) were used to trim low-quality base pairs from the 3′ end of each sequence and the quality of the raw and clean data was checked with FastQC (https://www.bioinformatics.babraham.ac.uk/projects/fastqc/ (accessed on 10 April 2024)). Syntenic gene blocks between SG1_hap1 and SG1_hap2 were identified using MCScanX with annotations and genomic sequences [58]. Gene pairs located in large gene blocks were further analyzed by reciprocal blastp, with alleles identified by more than 85% identity between protein sequences. To perform haplotype-resolved expression analysis, the assembly and annotations of SG1_hap1 and SG1_hap2 were combined into a metagenome. Quality-filtered RNA-seq reads were mapped to the metagenome using STAR [59] with the following parameters: outMultimapperOrder Random, outSAMtype BAM Unsorted, quantMode GeneCounts, alignIntronMax 6000, outSJfilter Reads Unique, and outFilterMismatchNmax 1 [60]. The alignments were sorted and filtered with SAMTools [61], and then uniquely mapped reads were obtained with Picard. Uniquely mapped genic reads were counted using StringTie to generate the expression matrix for all allele pairs. Genes were considered as expressed if the sum expression level of a pair of alleles (in TPM) was more than 1. A pair of bialleles of an expressed gene having a fold change in expression level ≥ 2 in at least one stage or tissue type was defined as biallele expression dominance (BED) [62].

To capture the dynamics of gene expression during lemongrass growth, co-expression network analysis was performed at the gene level using R package WGCNA [63,64]. An expression matrix of 18,352 expressed genes was used for block-wise co-expression network analysis. Briefly, the expression correlation between genes was calculated using a robust biweight-midcorrelation method [64] and raised to a soft threshold power, in which the co-expression network was fitted to a scale-free topology (Figure S5). Next, a signed-hybrid weighted correlation network was used to identify modules of interconnected genes with high topological overlap (TO). Co-expression modules were defined as branches of a hierarchical clustering tree, which was conducted using the dynamic tree cut method with a minimum module size of 30 genes. The expression patterns of each module were summarized as the module eigengenes. Pairs of closely related modules (module eigengene correlation >0.9) were merged. The transformation of expression correlation values to TO co-expression values effectively captured the relationships among neighborhoods of genes, making it a more robust and accurate approach than traditional clustering methods, which are simply based on similarities among gene expression.

### 3.6. qPCR-Based Expression Profiling of CfARFs

Total RNA was extracted from the leaf tissues of lemongrass seedlings using the plant total RNA kit (Zomanbio, Beijing, China), and cDNA was reverse transcribed with All-in-One RT Super-Mix (Vazyme, Nanjing, China). qRT-PCR was carried out using SYBR Green Master Mix (Vazyme, Nanjing, China) on a CFX96 real-time system (Bio-Rad, Hercules, CA, USA). *CfActin* (hap1.evm.model.Chr01.4294) was used as the internal reference gene for qPCR. The qPCR program included pre-denaturation at 95 °C for 10 min and 40 cycles of denaturation at 95 °C for 10 s, annealing at 60 °C for 30 s, and extension at 72 °C for 1 min. The primers used for qPCR are provided in Table S2.

### 3.7. Statistically Analysis

For the RNA-seq expression analysis, differentially expressed genes were calculated at the gene level by using the edgeR package (q value < 0.05 with the absolute value of expression fold change > 1). For the quantitative PCR analysis, statistical differences were determined by using Student’s *t*-test (*p* < 0.05). For the enrichment analysis of functional terms or ARF genes in a given co-expression module, the hypergeometric test was used (*P_hypergeometric_* < 0.05) with the ClusterProfiler package [65].

## 4. Conclusions

In this study, we provide several new and valuable findings regarding the CfARF family. (1) The genome-wide analysis provides a new catalog of CfARFs at the haplotype level as a starting point for understanding auxin-mediated gene expression regulation in lemongrass; (2) we demonstrate that WGD and TD both contribute to the expansion of CfARFs; (3) by combining detailed domain architecture analysis and expression profiling, our results indicate that differential expression may play a major role in driving the divergence of duplicated *CfARF* pairs, and variations in domains or motifs also contribute to the divergence of duplicated *CfARF*s; and (4) transcriptome analysis identified the candidate *CfARFs* and the associated molecular networks that could play a role during the growth of lemongrass. Particularly, our analyses suggest that activator *CfARF3* should play a role in leaf development, while activators *CfARF16* and *35* play a role in root development, with *CfARF9*, *18*, *27*, and *35* having functions in stem during growth. For repressor *CfARF*s in auxin-mediated gene expression, *CfARF10* and *CfARF7* could be involved in root growth. Thus, the functions of the above-mentioned *CfARF*s merit investigation in the future to test their roles in the auxin-mediated growth of lemongrass and potential for molecular breeding. In conclusion, this is, to the best of our knowledge, the first gene identification study using the haplotype-resolved genomes and transcriptome of lemongrass, which demonstrates haplotypic differences in *ARF* gene content, and it lays a foundation for future functional genomic studies of lemongrass and molecular breeding, with the goal of enhancing plant growth.

## Figures and Tables

**Table 1:** Information of the *CfARF* gene family.

CfARFs	Gene ID	Chr.	Hap.	Phylogenetic Clade	CDS Length (bp)	Prot. Length (aa)	MW (Da)	pI	DD1	B3	DD2	Q	LFG	III	IV	NCBI Accession
27	hap1.Chr10.1986	10	1	activator	3168	1055	263,067.26	4.81	√	√	√	√	√	√	√	PP763312
27	hap2.Chr10.2023	10	2	activator	3168	1055	262,997.19	4.81	√	√	√	√	√	√	√	PP763338
1	hap1.Chr07.2233	7	1	activator	3258	1085	270,104.51	4.81	√	√	√	√	√	√	√	PP763313
1	hap2.Chr07.267	7	2	activator	3258	1085	270,104.51	4.81	√	√	√	√	√	√	√	PP763339
9	hap1.Chr09.275	9	1	activator	2691	896	223,388.16	4.85	√	√	√	√	ACG	√	√	PP763314
9	hap2.Chr09.1852	9	2	activator	2691	896	223,342.08	4.85	√	√	√	√	ACG	√	√	PP763341
18	hap1.Chr10.582	10	1	activator	2754	917	229,989.87	4.83	√	√	√	√	√	√	√	PP763316
18	hap2.Chr10.586	10	2	activator	2754	917	230,160.25	4.83	√	√	√	√	√	√	√	PP763342
35	hap1.Chr10.288	10	1	activator	3495	1164	290,444.40	4.78	√	√	√	√	√	√	√	PP763317
35	hap2.Chr10.306	10	2	activator	3468	1155	288,415.02	4.78	√	√	√	√	√	√	√	PP763343
3	hap1.Chr08.2725	8	1	activator	2454	817	204,118.89	4.85	√	√	√	√	√	√	√	PP763321
3	hap2.Chr08.2730	8	2	activator	2175	724	180,852.58	4.89	P	√	√	√	√	√	√	PP763347
4	hap1.Chr08.2655	8	1	activator	2838	945	234,788.26	4.84	√	√	√	X	X	√	√	PP763323
4	hap2.Chr08.2661	8	2	activator	2838	945	234,788.26	4.84	√	√	√	X	X	√	√	PP763349
5a	hap1.Chr05.392	5	1	activator	2403	800	198,766.86	4.88	√	√	√	√	√	√	X	PP763329
5a	hap2.Chr05.404	5	2	activator	1242	413	102,288.83	5.02	√	√	√	X	X	P	X	PP763353
5b	hap1.Chr05.394	5	1	activator	3447	1148	285,393.94	4.79	√	√	√	√	√	√	√	PP763318
5b	hap2.Chr05.406	5	2	activator	3444	1147	285,142.60	4.79	√	√	√	√	√	√	√	PP763344
16	hap1.Chr05.549	5	1	activator	2739	912	228,594.64	4.84	√	√	√	√	√	√	√	PP763319
16	hap2.Chr05.549	5	2	activator	2739	912	228,566.59	4.84	√	√	√	√	√	√	√	PP763345
22	hap1.Chr02.3878	2	1	activator	426	141	35,663.89	5.24	X	X	P	X	X	X	X	PP763336
7	hap2.Chr03.4242	3	1	repressor	2079	692	169,965.69	4.91	√	√	√	X	√	√	√	PP763359
7	hap1.Chr03.45	3	2	repressor	2079	692	169,963.65	4.91	√	√	√	X	√	√	√	PP763332
8	hap1.Chr09.1160	9	1	repressor	2511	836	206,575.17	4.87	√	√	√	X	√	√	√	PP763324
8	hap2.Chr09.1023	9	2	repressor	2517	838	207,017.58	4.87	√	√	√	X	√	√	√	PP763350
10a	hap1.Chr03.3974	3	1	repressor	2430	809	200,683.10	4.86	√	√	√	X	√	√	√	PP763322
10a	hap2.Chr03.379	3	2	repressor	2430	809	200,729.19	4.86	√	√	√	X	√	√	√	PP763348
10b	hap2.Chr10.104	10	2	repressor	2559	852	212,894.83	4.85	√	√	√	X	√	√	√	PP763358
13	hap1.Chr04.1550	4	1	repressor	2565	854	209,962.57	4.86	√	√	√	X	√	√	√	PP763326
13	hap2.Chr04.1557	4	2	repressor	2565	854	210,020.65	4.86	√	√	√	X	√	√	√	PP763352
14	hap1.Chr05.1789	5	1	repressor	1977	658	163,664.80	4.92	√	√	√	X	√	√	√	PP763325
14	hap2.Chr05.1741	5	2	repressor	1977	658	163,708.85	4.92	√	√	√	X	√	√	√	PP763351
39	hap1.Chr08.1047	3	1	repressor	1986	661	164,430.61	4.92	√	√	√	X	√	√	√	PP763328
39	hap2.Chr08.1022	3	2	repressor	1986	661	164,462.67	4.92	√	√	√	X	√	√	√	PP763355
12	hap1.Chr03.2455	3	1	ETTN-like	2142	713	174,846.07	4.90	√	√	√	X	X	na	na	PP763331
12	hap2.Chr03.1907	3	2	ETTN-like	2163	720	176,745.27	4.90	√	√	√	X	X	na	na	PP763357
24	hap1.Chr06.300	6	1	ETTN-like	2127	708	175,645.46	4.88	√	√	√	X	X	na	na	PP763330
24	hap2.Chr06.302	6	2	ETTN-like	2223	740	183,107.48	4.88	√	√	√	X	X	na	na	PP763356
11	hap1.Chr03.2920	3	1	ETTN-like	2058	685	168,347.28	4.93	√	√	√	X	X	na	na	PP763334
11	hap2.Chr03.1440	3	2	ETTN-like	2049	682	167,593.36	4.93	√	√	√	X	X	na	na	PP763361
23	hap2.Chr06.618	6	1	ETTN-like	2052	683	167,136.26	4.92	√	√	√	X	X	na	na	PP763360
23	hap1.Chr06.605	6	2	ETTN-like	2052	683	167,182.35	4.92	√	√	√	X	X	na	na	PP763333
2	hap1.Chr01.3255	1	1	unknown	2073	690	169,779.02	4.88	√	√	√	X	√	√	√	PP763311
2	hap2.Chr01.2164	1	2	unknown	2073	690	169,524.45	4.88	√	√	√	X	√	√	√	PP763337
31a	hap1.Chr08.2898	8	1	unknown	1572	523	133,420.31	4.86	√	√	√	X	√	X	X	PP763335
31a	hap2.Chr08.2889	8	2	unknown	1566	521	132,723.33	4.87	√	√	√	X	√	X	X	PP763362
31b	hap2.Chr01.3181	1	2	unknown	558	185	45,378.47	5.20	√	√	P	X	√	X	X	PP763363
6	hap1.Chr08.1599	8	1	unknown	1830	609	152,317.09	4.84	√	√	√	X	√	P	X	PP763327
6	hap2.Chr08.1582	8	2	unknown	1764	587	146,798.63	4.85	√	√	√	X	√	P	X	PP763354
15	hap1.Chr05.2176	5	1	unknown	2121	706	175,252.12	4.81	√	√	√	X	√	√	√	PP763320
15	hap2.Chr05.2157	5	2	unknown	2121	706	175,238.09	4.81	√	√	√	X	√	√	√	PP763346
21	hap1.Chr10.490	10	1	unknown	2118	705	173,757.45	4.84	√	√	√	X	√	√	√	PP763315
21	hap2.Chr10.500	10	2	unknown	2154	717	176,664.72	4.83	√	√	√	X	√	√	√	PP763340

Notes: “Chr.” means chromosome number; “Hap.” means haplotype; “DD1” and ”DD2” mean dimerization domains 1 and 2, respectively; “B3” means the B3 DNA-binding domain; “Q” means the Q-rich stretch in the middle region of a ARF protein; “LFG” means the LFG motif, as previously described [14]; “III” and “IV” mean motifs III and IV in the C-terminal region of an ARF. For the conservation of each domain or motif, a checkmark means the domain or motif is conserved, while a cross mark means the domain or motif is not conserved, with “P” meaning that the domain or motif sequence is partially correct. “na” means “not applicable” and describes that ETTN-like ARFs usually have non-conserved C-terminal sequences and therefore checking the conservation of motifs III and IV for ETTN-like ARFs is not applicable.

## Data Availability

The data presented in the study are available in the article and the Supplementary Materials. For further inquiries, please contact the corresponding author directly.

## Supplementary Materials

The following supporting information can be downloaded at: https://www.mdpi.com/article/10.3390/ijms25158154/s1.
