# Supplementary material for: Divergent Roles of the Auxin Response Factors in Lemongrass (Cymbopogon flexuosus (Nees ex Steud.) W. Watson) during Plant Growth
Source: Int J Mol Sci. 2024 Jul 26;25(15):8154. doi: 10.3390/ijms25158154 (PMC11312390; doi:10.3390/ijms25158154)
Supplement: Supplementary file 1 [file ijms-25-08154-s001.zip › Yin_2024IJMS_FigS2.pdf]

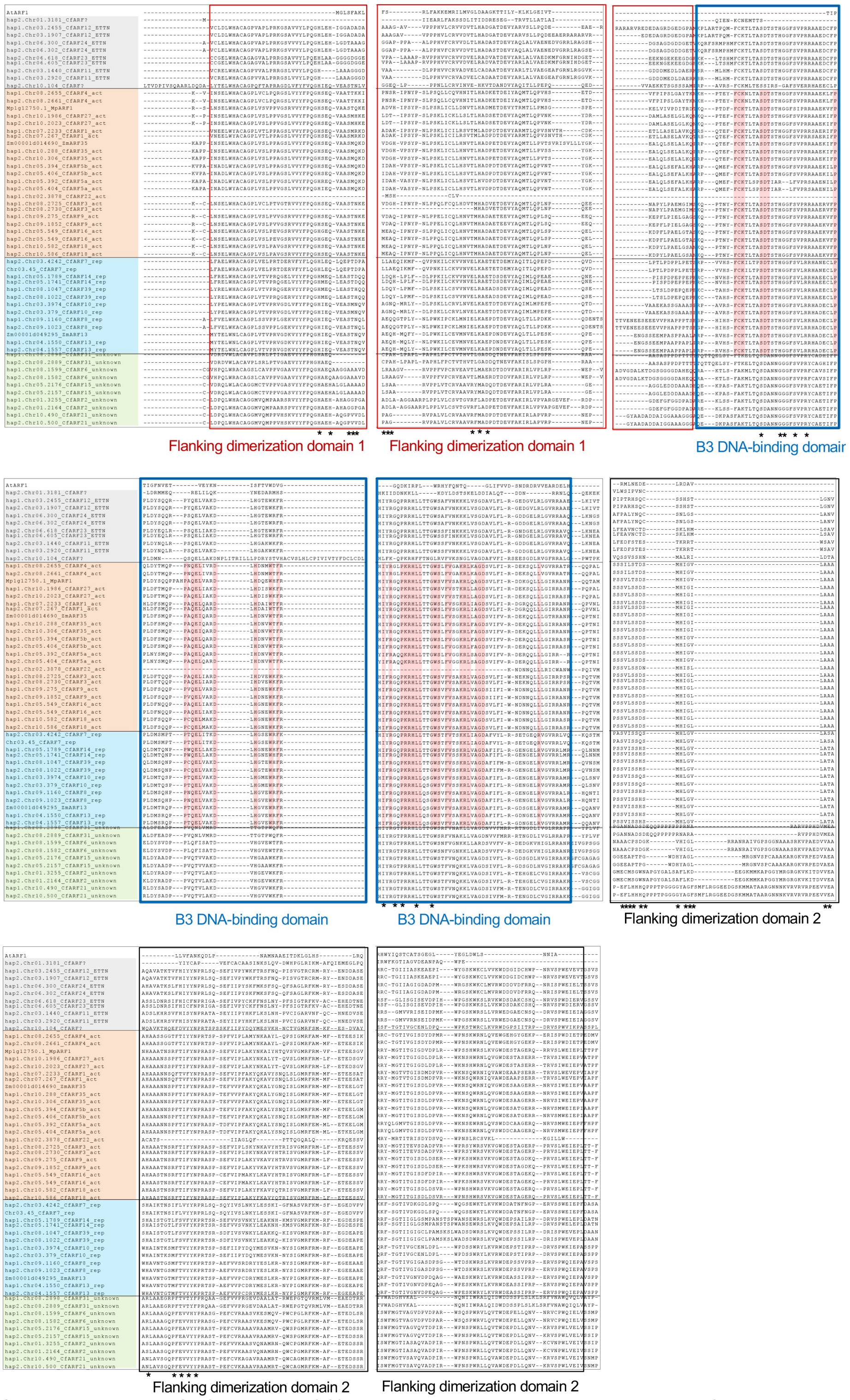


**Supplementary Figure S2** Conservation of CfARF DNA binding domain. Amino acid alignment of the DNA binding domain of CfARFs with representative activator and repressor ARFs (MpARF1 and ZmARF35 as the activators, AtARF1 and ZmARF13 as the repressors) were performed. The phylogenetic clades for CfARFs are indicated in background colors (orange, light blue, grey and green meaning group 1, 2, 3, and 4, respectively). B3 DNA-binding domain (DBD) is indicated by the blue box, while flanking dimerization domain 1 and 2 are indicated by the red and grey boxes, respectively. The sequences of flanking dimerization domains 1 and 2 were adopted from those described previously (Boer 2014; Guilfoyle 2015; Galli 2018). Asterisks in the B3 DBD region indicate DNA contacting residues described for AtARF1 (Boer 2014). Asterisks in the flanking dimerization domains indicate residues at the ARF dimer interface (Galli 2018). Amino acid residues conserved in the aligned ARFs for either group-1 or group-2 ARFs are highlighted with red background color.
