# Supplementary material for: Divergent Roles of the Auxin Response Factors in Lemongrass (Cymbopogon flexuosus (Nees ex Steud.) W. Watson) during Plant Growth
Source: Int J Mol Sci. 2024 Jul 26;25(15):8154. doi: 10.3390/ijms25158154 (PMC11312390; doi:10.3390/ijms25158154)
Supplement: Supplementary file 1 [file ijms-25-08154-s001.zip › Yin_2024IJMS_FigS3.pdf]

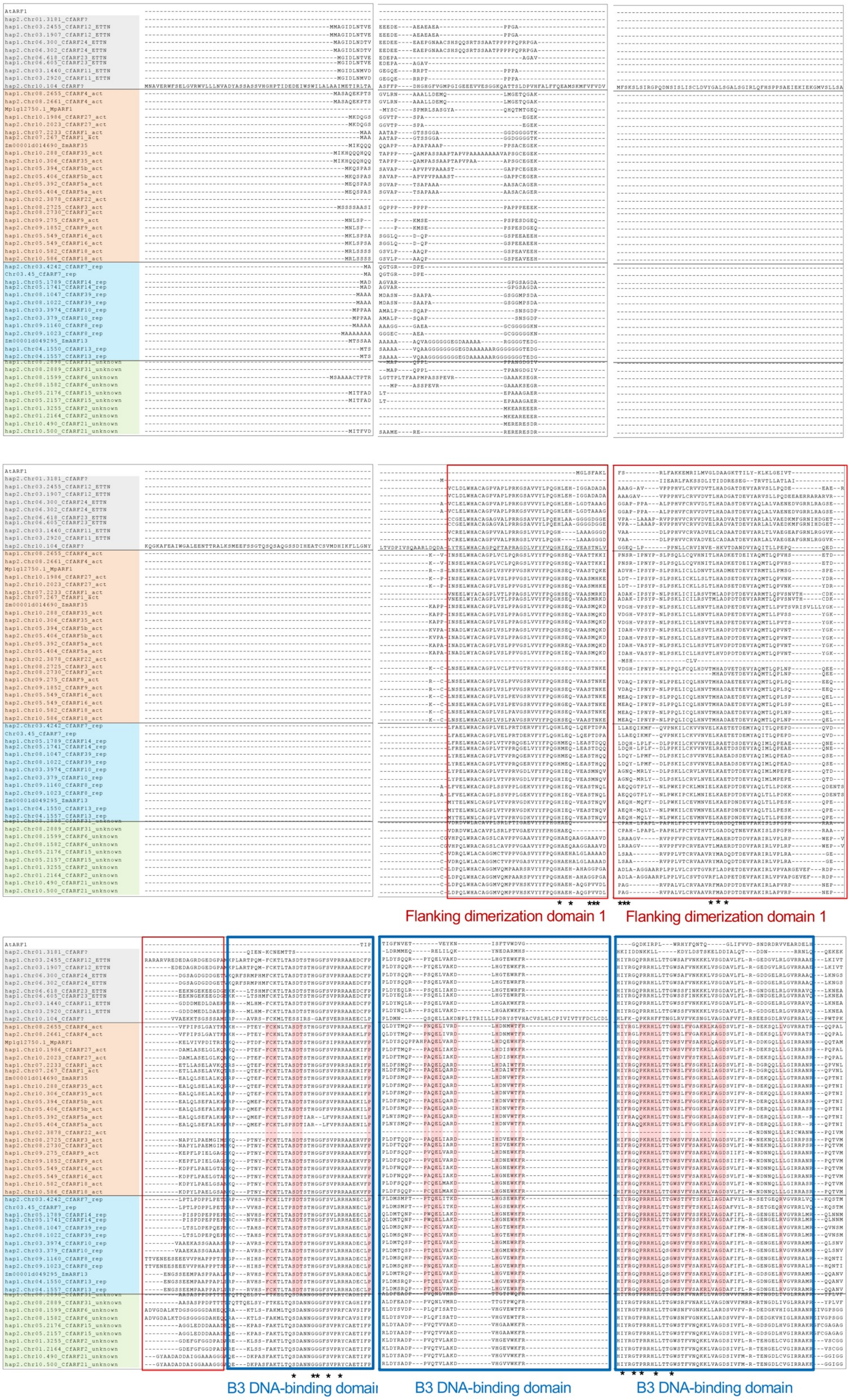
Flanking dimerization domain 1
Flanking dimerization domain 1
B3 DNA-binding domai
B3 DNA-binding domain
B3 DNA-binding domain

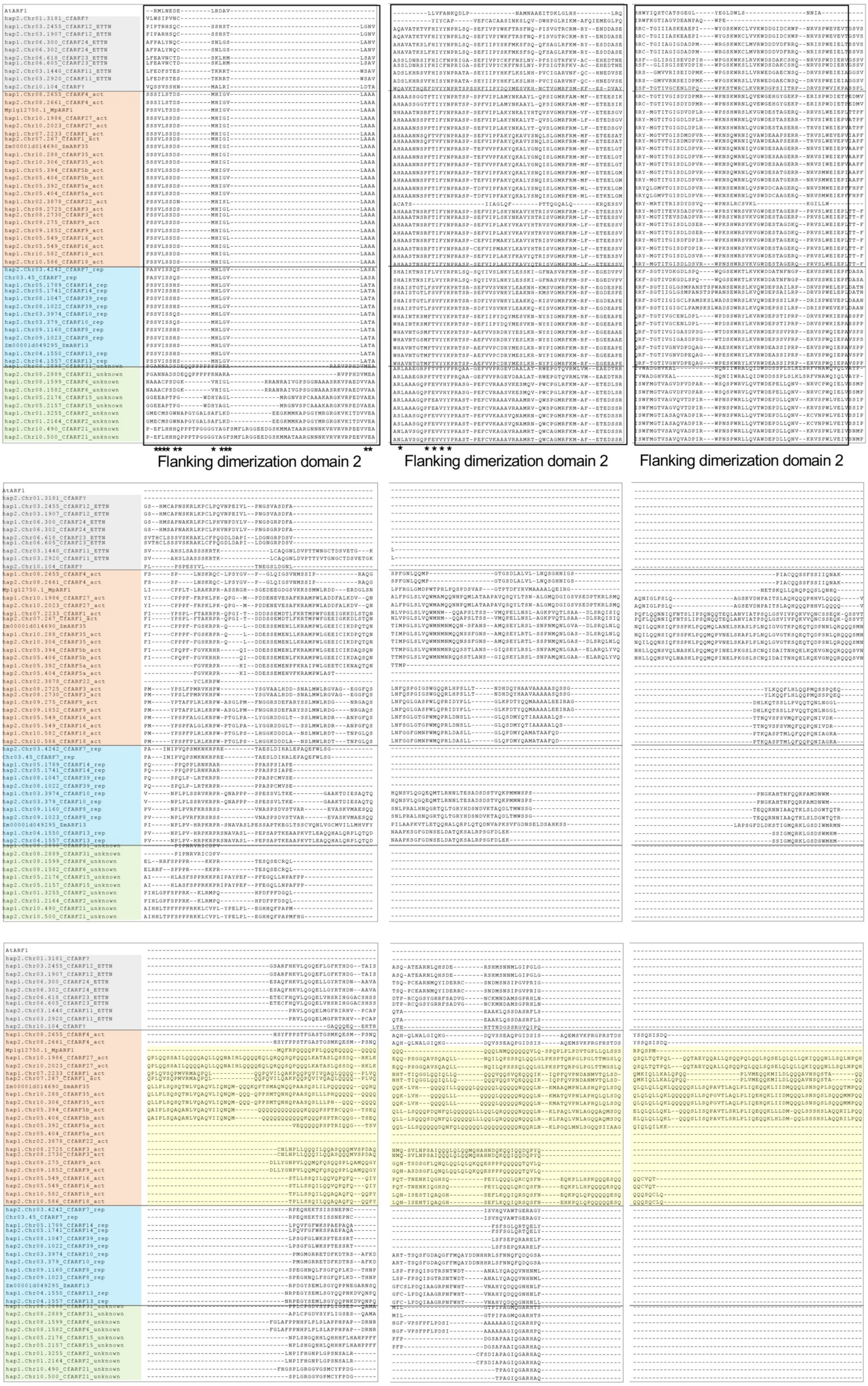
Flanking dimerization domain 2
Flanking dimerization domain 2
Flanking dimerization domain 2

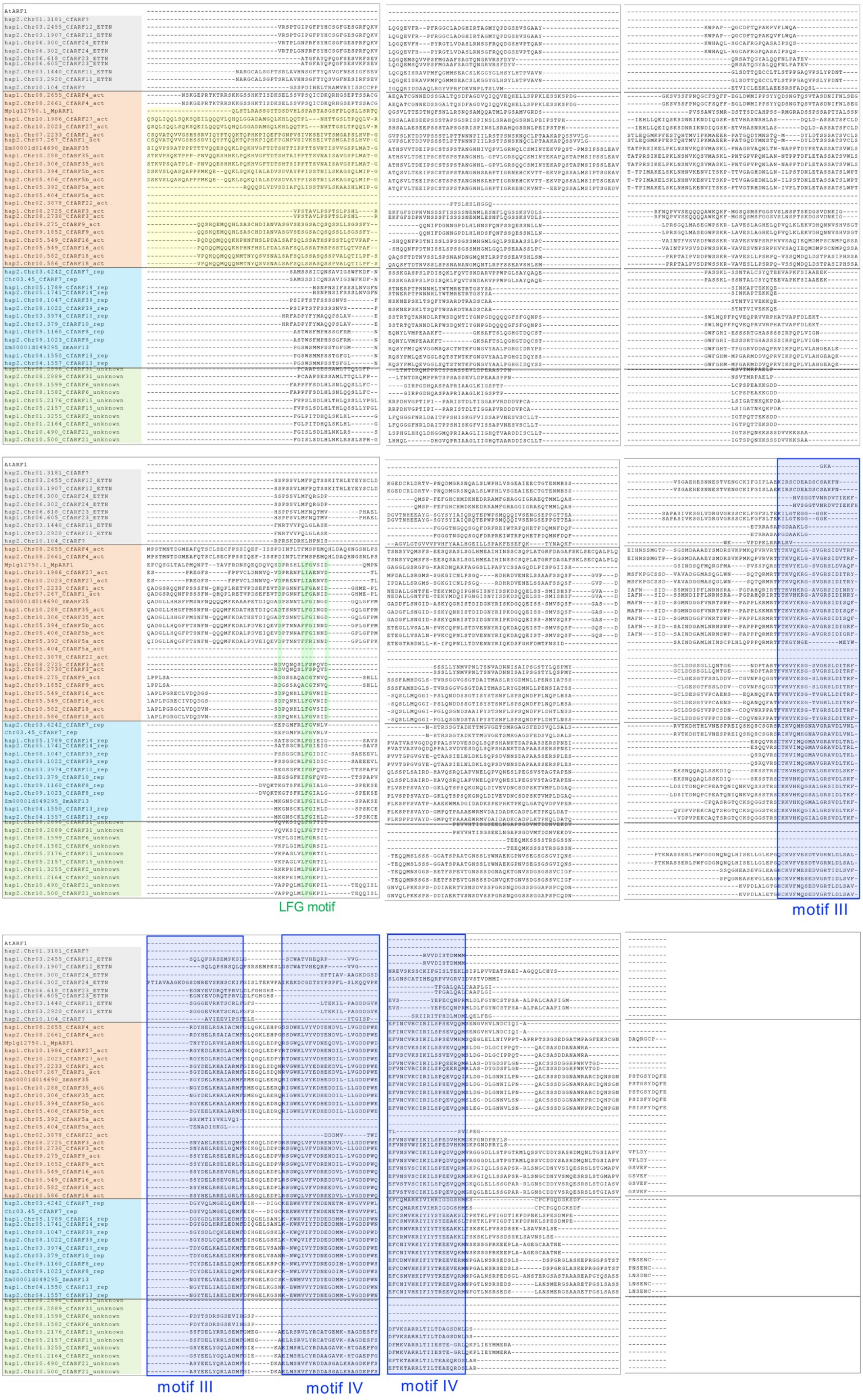


**Supplementary Figure S3** Full-length protein sequence alignment between CfARFs.

Alignment of the full-length protein sequences of CfARFs with representative activator and repressor ARFs (MpARF1 and ZmARF35 as the activators, AtARF1 and ZmARF13 as the repressors) were performed. The phylogenetic clades for CfARFs are indicated in background colors (orange, light blue, grey and green meaning group 1, 2, 3, and 4, respectively). B3 DNA-binding domain (DBD) is indicated by the blue box, while flanking dimerization domain 1 and 2 are indicated by the red and grey boxes, respectively. The sequences of flanking dimerization domains 1 and 2 were adopted from those described previously (Boer 2014; Guilfoyle 2015; Galli 2018). Asterisks in the B3 DBD region indicate DNA contacting residues described for AtARF1 (Boer 2014). Asterisks in the flanking dimerization domains indicate residues at the ARF dimer interface (Galli 2018). Amino acid residues conserved in the aligned ARFs for either group-1 or group-2 ARFs are highlighted with red background color. In pages 2 and 3 of this file, the Q stretches specific for the activator ARFs are labeled with light yellow background.

The amino acid sequences similar to Aux/IAA motif III or IV are indicated in blue boxes, while the LFG motif specific for the activator ARFs is indicated in light green background.
