# Supplementary material for: Divergent Roles of the Auxin Response Factors in Lemongrass (Cymbopogon flexuosus (Nees ex Steud.) W. Watson) during Plant Growth
Source: Int J Mol Sci. 2024 Jul 26;25(15):8154. doi: 10.3390/ijms25158154 (PMC11312390; doi:10.3390/ijms25158154)
Supplement: Supplementary file 1 [file ijms-25-08154-s001.zip › Yin_2024IJMS_Suppl20240722.pdf]

International Journal of
Molecular Sciences

MDPI

Supplementary Information for

Divergent Roles of the Auxin Response Factors in Lemongrass (*Cymbopogon flexuosus* (Nees ex Steud.) W. Watson) during Plant Growth

Guoli Wang[1,2,#], Jian Zeng[1,#], Canghao Du[2], Qi Tang[2], Yuqing Hua[3], Mingjie Chen[2], Guangxiao Yang[2], Min Tu[3], Guangyuan He[2], , Yin Li[2], Jinming He[1,*], Junli Chang[2,*]

1 Guangdong Provincial Key Laboratory of Utilization and Conservation of Food and Medicinal Resources in Northern Region, School of Biology and Agriculture, Shaoguan University, Shaoguan, Guangdong, 512005, China;
2 The Genetic Engineering International Cooperation Base of Chinese Ministry of Science and Technology, Key Laboratory of Molecular Biophysics of Chinese Ministry of Education, College of Life Science and Technology, Huazhong University of Science and Technology, Wuhan 430074, China;
3 Hubei Technical Engineering Research Center for Chemical Utilization and Engineering Development of Agricultural and Byproduct Resources, School of Chemical and Environmental Engineering, Wuhan Polytechnic University, Wuhan 430023, China.
# These authors contributed equally to this work.
* Correspondence: cjl@hust.edu.cn (J.C.); jmh-3183@163.com (J.H.)

The supplementary information of this manuscript contains:

Supplementary Table S1. Expression matrix of the RNA-seq dataset; (Provided in a separate EXCEL file)

Supplementary Table S2. qPCR primers used in the study;

Supplementary Table S3. Functional enrichment results of the 21 co-expression modules; (Provided in a separate EXCEL file)

Supplementary Figure S1. Protein identity matrix between CfARF proteins;

Supplementary Figure S2. Protein sequence alignment of the DNA binding domain between CfARFs; (Provided in a separate PDF file)

Supplementary Figure S3. Full-length protein sequence alignment between CfARFs; (Provided in a separate PDF file)

Supplementary Figure S4. Samples used for transcriptome analysis;

Supplementary Figure S5. Generation of co-expression network with WGCNA.

Supplementary Figure S6. Comparison of the expression patterns of four whole-genome duplicated OsARF pairs.

Supplementary Table S2 qPCR primers used in the study.

Primer Name	Sequence (5′-3′)	Tm (◦C)	Purpose	Product (bp)
ARF3_F	ATGGGCTCACAATCCATGTCTT	60	qPCR analysis for CfARF3	138 bp
ARF3_R	GGAGGAAGAGTCAACTTGAGGG	60		
ARF10_F	TGGAAGATTGAGCCTGCTTCAT	60	qPCR analysis for CfARF10	208 bp
ARF10_R	TGTTCCTCAAGGTCATCTGCTC	60		

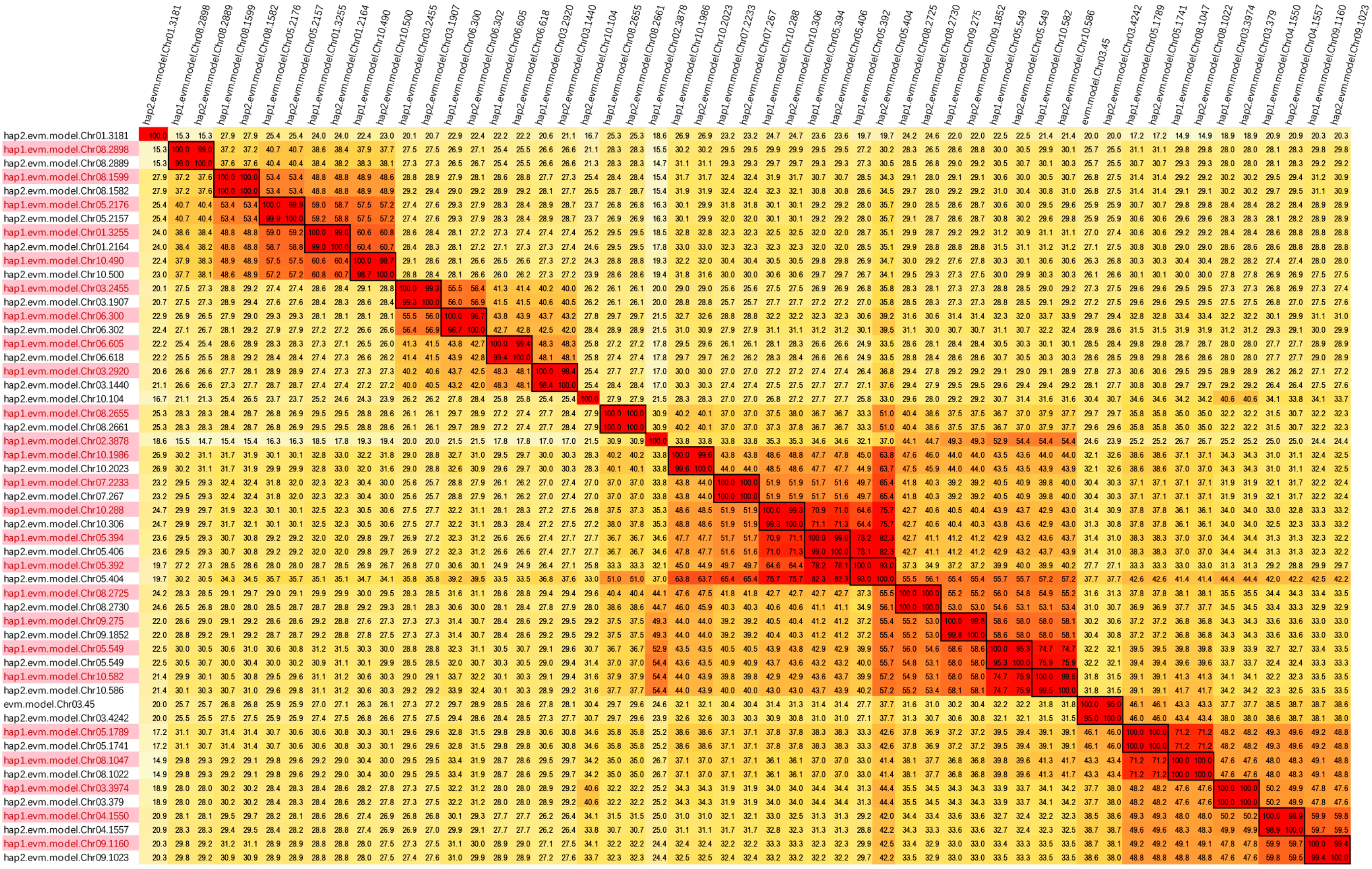

Supplementary Figure S1 Protein identity matrix between CfARF proteins. The color indicates the identity of each pair of protein sequences of CfARFs, with the protein identities between haplotypic CfARFs are shown in black boxes.

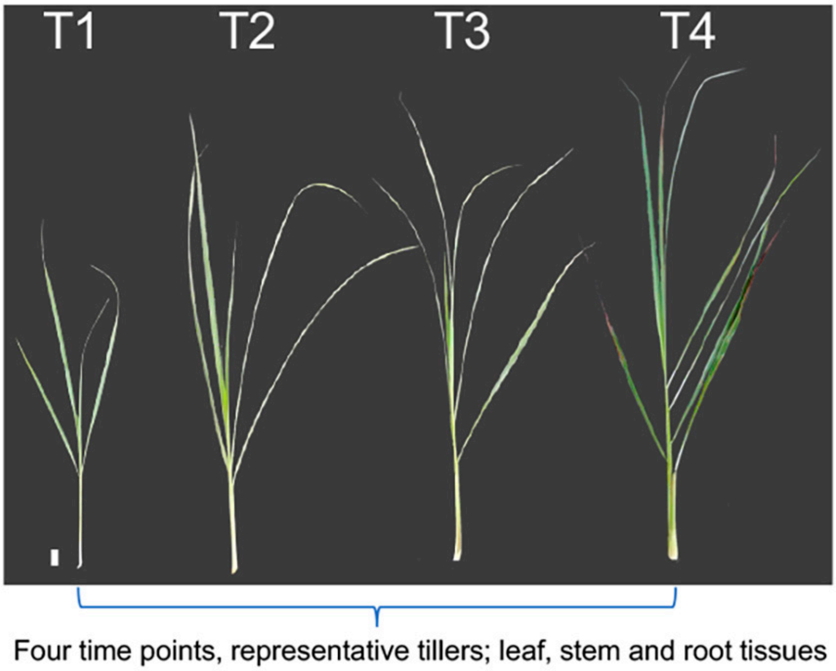

Omics	Leaf tissue	Stem tissue	Root tissue
transcriptomics	✓	✓	✓

Supplementary Figure S4. Samples used for transcriptome analysis. Representative tillers at the T1, T2, T3, and T4 stages are shown.

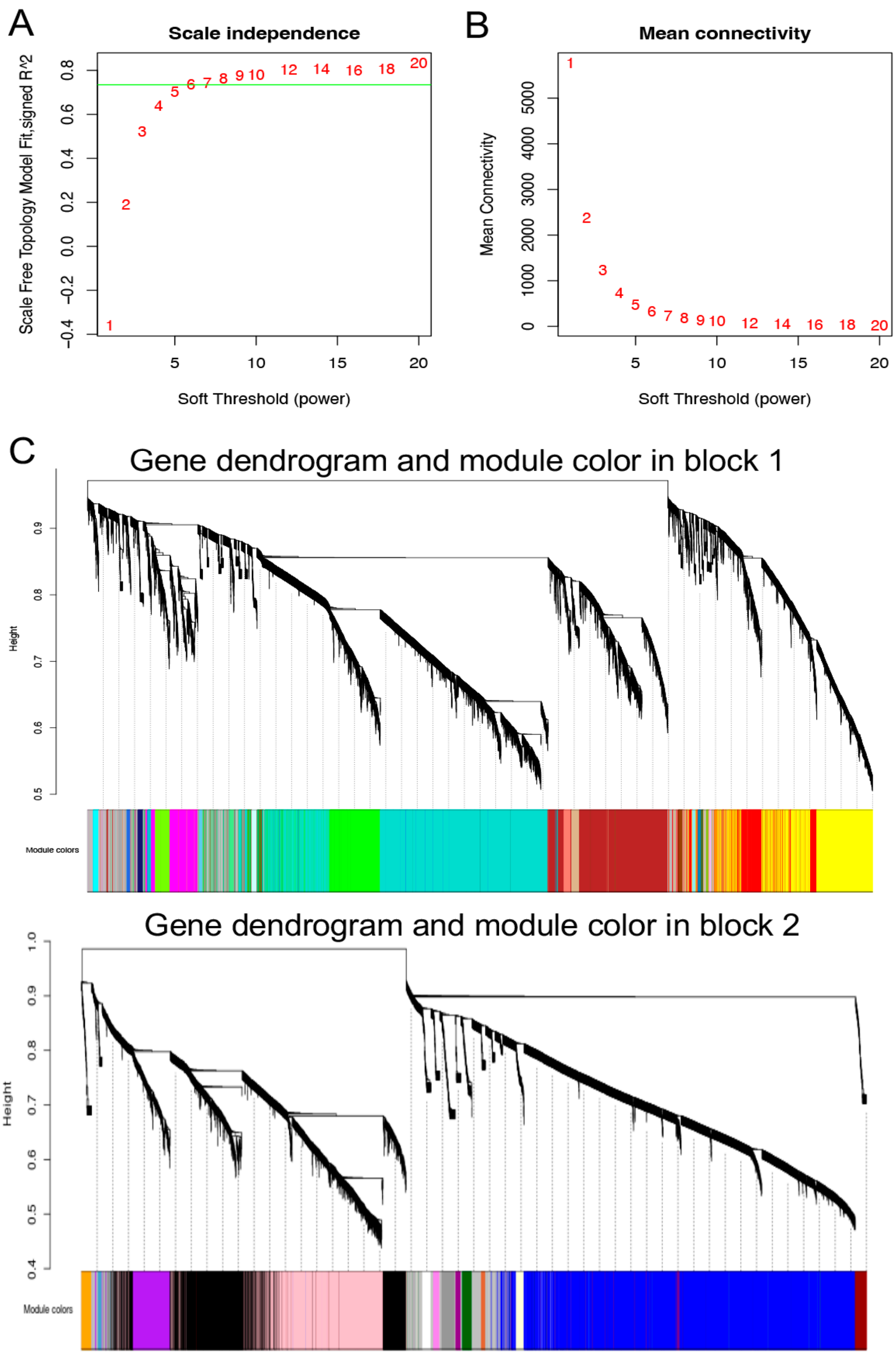

Supplementary Figure S5. Generation of co-expression network with WGCNA. (A and B) Selection of soft threshold (power) for the construction of weighted gene co-expression network. After raising the co-expression network to a power threshold, the network topology is approximately scale-free, with a coefficient of determination to scale-free topology being over 0.75; (C) The gene dendrogram and module assignment with the block-wise construction of weighted gene co-expression network analysis.

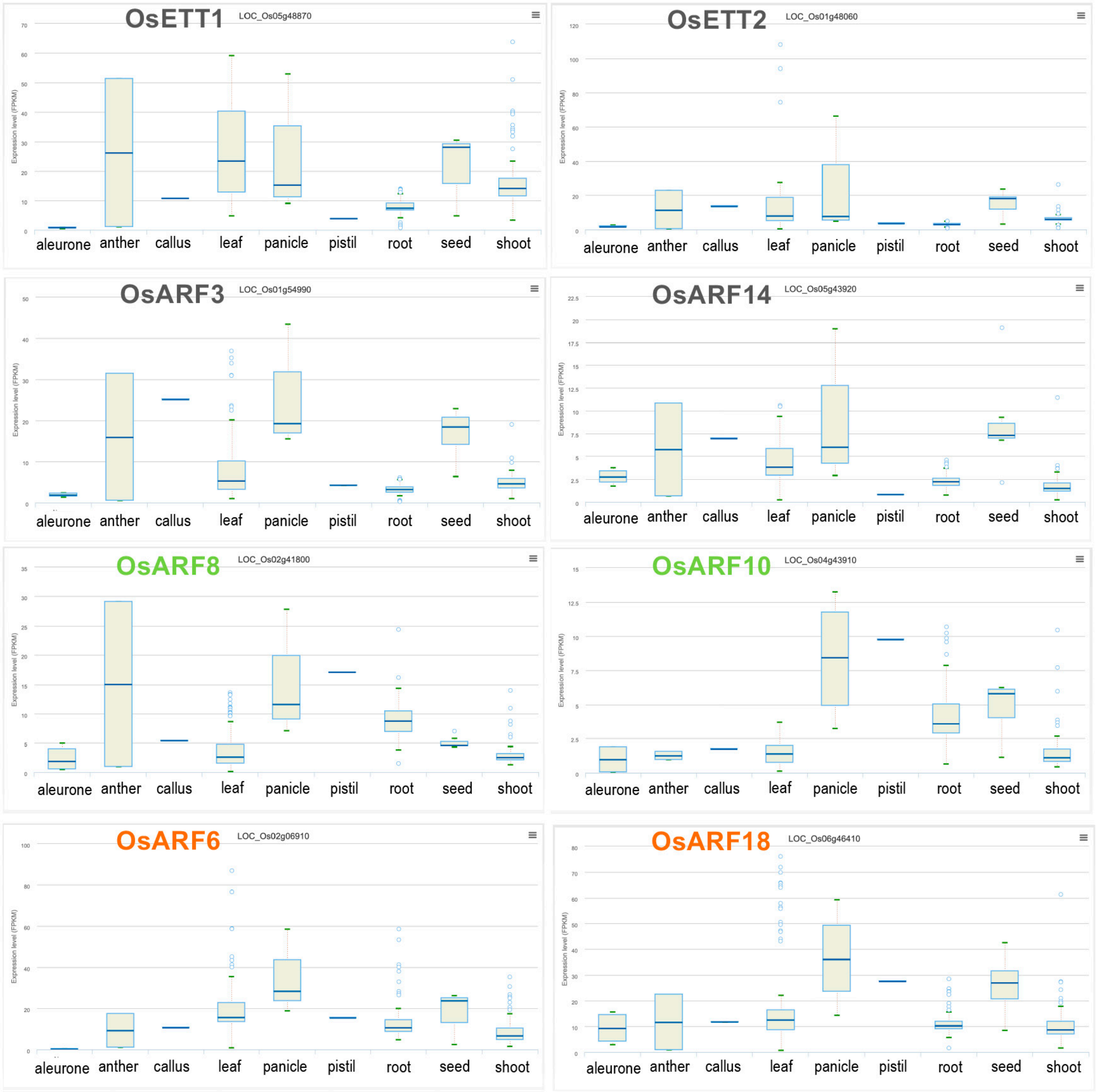

Supplementary Figure S6. Comparison of the expression patterns of four whole-genome duplicated OsARF pairs. The expression patterns were obtained from the IC4R expression database (Sang et al., 2020)

References:

Sang, J., Zou, D., Wang, Z., Wang, F., Zhang, Y., Xia, L., Li, Z., Ma, L., Li, M., Xu, B., Liu, X., Wu, S., Liu, L., Niu, G., Li, M., Luo, Y., Hu, S., Hao, L., Zhang, Z. IC4R-2.0: Rice Genome Reannotation Using Massive RNA-seq Data. *Genom. Proteo. Bioinfor*. **2020**, *18*, 161-172.
